# Identification and Analysis of Multi-Protein Complexes in Placenta

**DOI:** 10.1371/journal.pone.0062988

**Published:** 2013-04-29

**Authors:** Fuqiang Wang, Ling Wang, Zhiyang Xu, Gaolin Liang

**Affiliations:** 1 CAS Key Laboratory of Soft Matter Chemistry, Department of Chemistry, University of Science and Technology of China, Hefei, Anhui, China; 2 State Key Laboratory of Reproductive Medicine, Analysis Center, Nanjing Medical University, Nanjing, Jiangsu, China; Brandeis University, United States of America

## Abstract

Placental malfunction induces pregnancy disorders which contribute to life-threatening complications for both the mother and the fetus. Identification and characterization of placental multi-protein complexes is an important step to integratedly understand the protein-protein interaction networks in placenta which determine placental function. In this study, blue native/sodium dodecyl sulfate polyacrylamide gel electrophoresis (BN/SDS-PAGE) and Liquid chromatography-tandem mass spectrometry (LC-MS/MS) were used to screen the multi-protein complexes in placenta. 733 unique proteins and 34 known and novel heterooligomeric multi-protein complexes including mitochondrial respiratory chain complexes, integrin complexes, proteasome complexes, histone complex, and heat shock protein complexes were identified. A novel protein complex, which involves clathrin and small conductance calcium-activated potassium (SK) channel protein 2, was identified and validated by antibody based gel shift assay, co-immunoprecipitation and immunoﬂuorescence staining. These results suggest that BN/SDS-PAGE, when integrated with LC-MS/MS, is a very powerful and versatile tool for the investigation of placental protein complexes. This work paves the way for deeper functional characterization of the placental protein complexes associated with pregnancy disorders.

## Introduction

The placenta plays a pivotal role of promoting the exchange of nutrients and waste products between the maternal and fetal circulatory systems [1]. In addition, it is a natural barrier against numerous bacterial and viral infections during pregnancy. Maternal preeclampsia (PE) and fetal intrauterine growth restriction (IUGR) are two of the most common and serious complications of human pregnancy associated with placental abnormalities [2], [3]. Each of these two disorder affects about 5% of all pregnancies [4]. As we known, development and functionalization of placenta are mediated by various proteins which have been investigated in proteomics and disciplines associated. Those studies have identified a number of abnormally expressed proteins in plasma, amniotic fluid, intact placentae, or trophoblasts from pre-eclampsia patients using various proteomic techniques including classical two dimensional (2D) gel electrophoresis, Isobaric tags for relative and absolute quantitation (iTRAQ), and Difference gel electrophoresis (DIGE) [5]. Recently, Cox *et al* developed a proteomics method to identify proteins from the blood tissue interfaces of placentas using intra-vascular silica-bead perfusion and shotgun proteomic analysis [6]. In their work, 1,181 plasma membrane proteins were identified, of which 171 were enriched at the maternal blood-trophoblast interface. Robinson *et al* reviewed the employment of proteomic techniques to exploit novel proteins in placenta which gives insights into placental biology [7]. Despite extensive researches having been done, the molecular mechanisms underlying placental function remain unclear. To date, most of the reported proteomic analyses concentrate on the protein expression profile within normal or diseased conditions of placentas [7]–[9]. They are not able to provide information about how these proteins interact with each other.

It has been proposed that most biological processes are performed by protein complexes [10]. For example, most cellular processes require several enzymes, which are usually associated with each other, to function together and form larger temporary or stable protein complexes for raising the efficiency, specificity and speed of metabolic pathways [11]. Therefore, identifying the composition of the placenta protein complexes will result in more abundant information of the function of placenta than that protein identities alone are able to deliver.

There are many ways to investigate protein interactions, such as two-step affinity purification [12], immunoprecipitations [13], or comprehensive two-hybrid screens [14]. Each method has its individual advantages and drawbacks. These approaches allow the detection of individual protein-protein interactions and investigation of the actual or possible interaction partner(s) of a particular protein of interest, but they are not designed to provide a whole view of protein-protein interaction in a complex proteome of choice within a single experiment.

Blue native PAGE (BN-PAGE) can be used for one-step isolation of protein complexes from biological membranes, total cells, or tissue homogenates. The principles of this method have been detailedly described by Hermann Schagger [15]. This technique offers the unique advantage of separating native protein complexes in biological samples with the samples maintaining undissociated. Moreover, the resolution of BN-PAGE is much higher than that of other methods such as gel filtration or sucrose-gradient ultracentrifugation [15], [16]. Integrating with MS, BN-PAGE has the potential to identify intact protein complexes which are either water soluble or insoluble (e.g., membrane proteins). BN-PAGE has been successfully used to screen protein complexes of synaptic plasma membrane, salt-induced halo tolerant alga, and so on [17], [18]. Nevertheless, to our best of knowledge, using BN-PAGE to separate protein complexes of placenta has not been reported. In this work, we report using BN-PAGE to identify and characterize a number of functional protein complexes from placenta, which supplements a new methodology for the proteomic analysis of placenta.

## Materials and Methods

### Sample Preparation

Placenta tissues were taken from twenty pregnant women according to the standard operating procedure. All of the mothers had cesarean section delivery in Maternal and Child Health Hospital of Nanjing and had same age range and gestational weeks. All the mothers provided written informed consent. The study was approved by the Ethics Committee of Nanjing Medical University with an Institutional Review Board (IRB) number of 2012-NFLZ-32. The detailed patient characteristics are presented in Table S1. To determine the optimal concentration of detergent for solubilizing protein complexes, we tested five different concentrations of Triton X-100 (0.25%, 0.5%, 0.75%, 1.5%, and 3%) and analyzed their solubilization efficiencies with SDS-PAGE and BN-PAGE.

### BN/SDS-PAGE

BN-PAGE was performed as described [16]. All buffers were adjusted to pH 7.0 at 4°C. 100 µg aliquots of placenta protein suspension were pelleted, resuspended in 20 µL mixtures of 50 mM NaCl, 5 mM 6-aminocaproic acid, 10% glycerol, 50 mM imidazole at pH 7.0, and five different concentrations (0.25%, 0.5%, 0.75%, 1.5%, and 3%) of Triton X-100 (sigma) and then incubated on ice for 30 min. After centrifugation, 3 µL 6-aminohexanoic acid at 750 mM with 5% (w/v) Coomassie Brilliant Blue G-250 was added into the total supernatants and then the mixture was loaded on a 4–13% blue native PAGE overlaid with a 3.5% stacking gel (0.15×1 cm gel wells). Electrophoresis was started at 100 V and 4°C until the samples completely entering the separating gel. The electrophoresis was subsequently continued at 4°C and voltage at 200 V until the dye front reached the gel bottom. The gel was stained with Coomassie G-250 after being removed from the cassette.

For further separation with 2D SDS-PAGE, the excised lanes (1.5% Triton X-100 treated) from BN-PAGE were denatured in 1% SDS with 1% β-mercaptoethanol for 3 h, placed into the SDS-PAGE cassette and sealed with hot agarose solution. Electrophoresis was performed at 25 mA until the sample front passed into the separation gel and then continued at 50 mA. Proteins were visualized after the gel was stained with a Commassie Blue Staining Kit (Beyotime Institute of Biotechnology, China).

For native antibody-based gel-shift assay, 5 µg of the anti-small conductance calcium-activated potassium (SK) channel protein antibody (Abcam, UK) were added to the samples (30 µL for each), mixed and incubated for 30 min on ice before being loaded on BN-PAGE.

### LC–MS/MS Analysis

9 bands from one dimensional (1D) BN-PAGE and 97 spots from 2D SDS-PAGE were cut off and then in-gel digestion was performed as described [19]. The dried extracted peptides were analyzed by LC-MS/MS. An Agilent 1100 series liquid chromatography system equipped with ZORBAX SB-C18 column (0.3 mm×150 mm, 5 µm) was used, with the mobile phases A and B being water/0.1% formic acid and acetonitrile/0.1% formic acid respectively. The flow rate was 200 nL/min. A gradient of mobile phase B was created according to the following scheme: 0–5 min, 0%; 5–65 min, from 0 to 60%; 65–70 min, from 60 to 100%; 70–80 min, 100%; 80–90 min, from 100 to 0%. Linear trap quadrupole Orbitrap (LTQ-Orbitrap) was operated in a data-dependent mode. Each full MS scan was followed by three MS^2^ scans. Turbo Sequest V2.7 software was used for data analysis. Peptide identification standard was set as following: Xcorr >2.5 when m/z is +2; Xcorr >3.0 when m/z is +3.

### Co-Immunoprecipitation

Placenta protein suspensions were pelleted and solubilized with 1.5% Triton X-100 in solution A (50 mM NaCl, 50 mM Tris, 1 mM EDTA and cocktail, pH 7) on ice for 1 h. After centrifugation, the supernatant protein (1 mg) was precleared with protein A-Sepharose for 20 min on ice and then incubated with appropriate immobilized affinity-purified mouse anti-clathrin (Abcam) or preimmunization IgG pool (negative control) overnight at 4°C. Subsequently, 20 µL of protein A was added, vortexed for 1 h at room temperature. After being washed with 0.1% Triton X-100 in solution A for three times, bound proteins were eluted with SDS loading buffer, then subjected to SDS-PAGE for separation and western blot for analysis.

### Immunofluorescence Staining

For immunolocalization of clathrin and SK channel protein 2, placenta sections (6 µm) were obtained as described and fixed with 4% (w/v) paraformaldehyde in PBS for 10 min [20]. Then the slides were washed with PBS, blocked in 2% BSA solution for 30 min at 37°C and incubated with primary antibodies for 1.5 h at room temperature. The primary antibodies were rabbit anti- SK channel protein 2 (Abcam; 1∶200) and mouse anti-clathrin (Abcam, 1∶200). Excess antibodies were removed by incubation of the tissue slides with 0.1% Tween-20 in PBS for 15 min. Tissues conjugated with first antibodies were then incubated with Alexa 594 goat anti-mouse and Alexa 488 goat anti-rabbit secondary antibodies. After 3 washes with PBS, the placenta tissues were imaged under a Zeiss Axioskop plus fluorescence microscope (Carl Zeiss, Jena, Germany), and the digital images were analyzed with AxioVision 3.1 software (Carl Zeiss, Jena, Germany).

## Results

### Protein Solubilization

**Figure 1:**
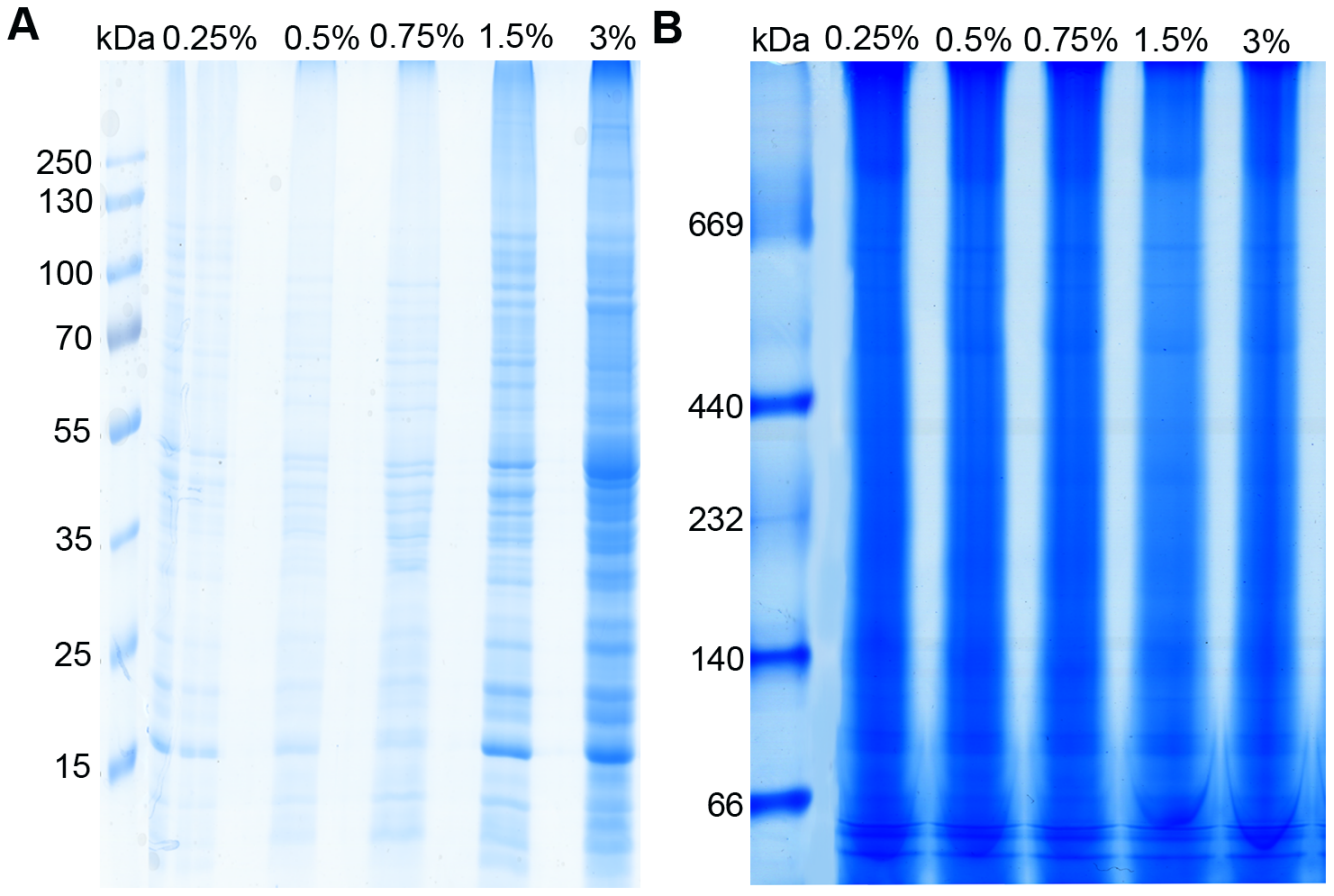
Blue native PAGE analysis of the placenta lysates. (A) The solubility of placenta proteins in native sample buffer was tested. After solubilization in varying concentrations of Triton X-100 (0.25%–3%), placenta lysates were subjected to SDS-PAGE. (B) Blue native PAGE analysis of the placenta lysates solubilized with Triton X-100 solutions at different concentrations (0.25%–3%).

To determine the optimal conditions for the solubilization of intact placenta protein complexes, a series of Triton X-100 concentrations were evaluated. Solubilization efficiencies of protein complexes under different concentrations of Triton X-100 were analyzed with SDS-PAGE (Figure 1A).The result indicates that 3% Triton X-100 solubilizes the protein more completely than other four concentrations of Triton X-100 do. Further characterization of the solubilized protein complexes was achieved with BN-PAGE (Figure 1B). The result indicates that the best separation effect is achieved with 1.5% Triton X-100. Therefore, in consideration of both the yield and separation of the protein complexes, 1.5% Triton X-100 was used in all following experiments for the solubilization of the intact placenta protein complexes.

### Overview of Placenta Proteins Identified by BN/SDS-PAGE

**Figure 2:**
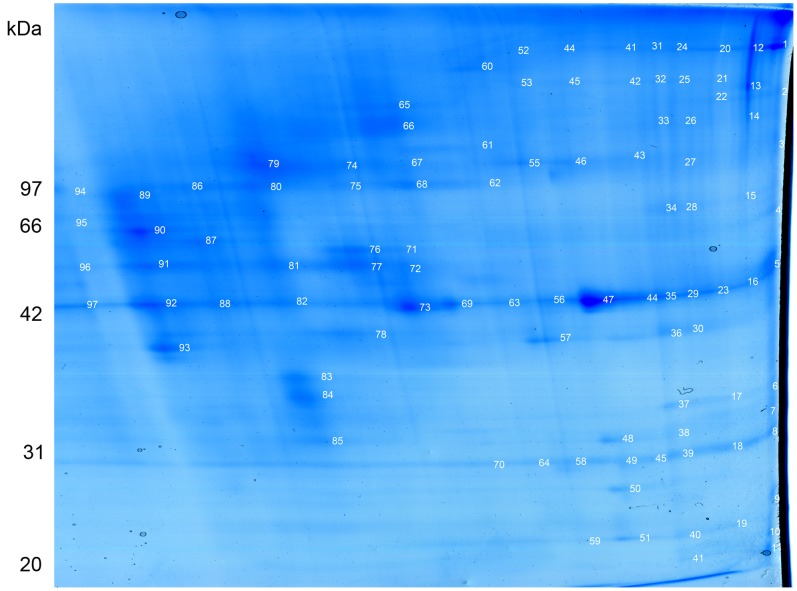
2D BN/SDS-PAGE proteomic maps of placental protein complexes solubilized with 1.5% Triton X-100. 1D BN strip (1.5% Triton X-100) in Figure 1B was loaded and separated on 11.5% acrylamide SDS-PAGE. The 1D BN strip was oriented with top to the left and bottom to the right. The proteomic map presented here is a representative coomassie brilliant blue (CBB)-stained protein gel. The gel spots for mass spectrometry identification were labeled with numbers.

BN-PAGE was used to separate proteins and protein complexes of placenta (Figure 1B). Nine distinct bands from 1.5% Triton X-100 treated lane in Figure 1B were excised, subjected to trypsin digestion, and MS analysis in turn. For separation on the second dimension, BN gel strip was loaded horizontally on the SDS-PAGE gel. Figure 2 shows a representative 2D BN/SDS-PAGE map of placenta protein complexes. Ninety-seven individual protein spots, which were consistently stained by Coomassie blue (labeled in Figure 2) were excised, digested with trypsin, and the recovered peptides were sequenced with nanoLC-MS/MS analysis. Using mascot database-searching algorithm, we identified 733 non-redundant proteins from the gel bands and spots in Figure 1B and Figure 2. All of the identified proteins were listed in Table S2 and their potential subcellular localization and biological functions were analyzed thereafter.

**Figure 3:**
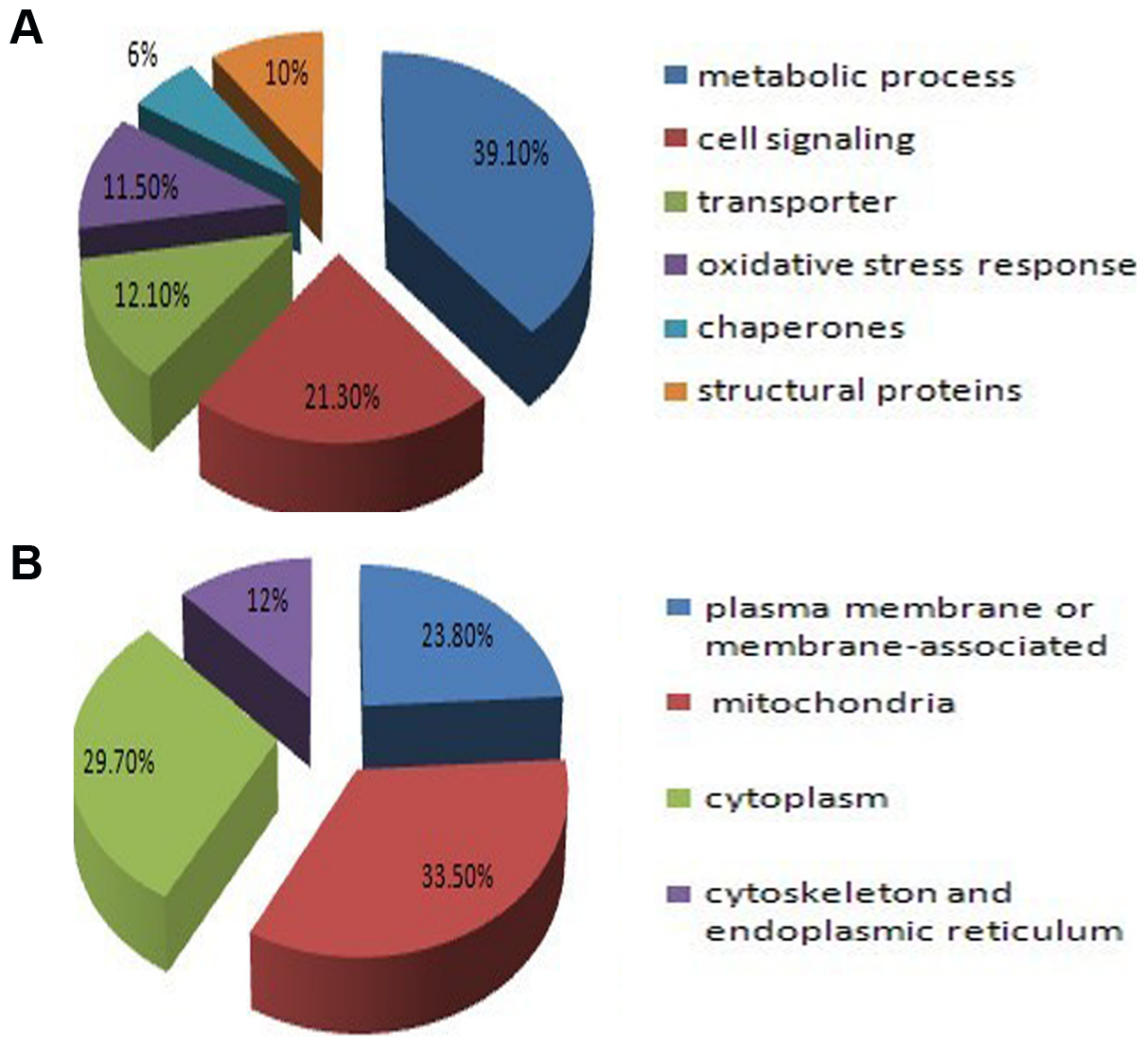
The functions and subcellular locations of the identified proteins from BN/SDS-PAGE. (A) The functions of the identified proteins according to the GO annotations and literatures. (B) The subcellular locations of the identified proteins according to the GO annotations and literatures.

The subcellular locations were cataloged according to the gene ontology (GO) component annotations from literatures. From Figure 3A, it is of interest to point out that proteins involved in metabolic process representing the largest functional category (39.1%) among the 733 proteins with different molecular functions including: cell signaling (21.3%), transporter (12.1%), oxidative stress response (11.5%), chaperones (6%), and structural proteins (10%). Figure 3B shows that approximately 23.8% of the identified proteins are plasma membrane or membrane-associated proteins. 33.5% proteins locate in mitochondria and 29.7% proteins locate in the cytoplasm. Other 12% proteins are mainly from cytoskeleton and endoplasmic reticulum.

### Placenta Protein Complexes Identified by BN/SDS-PAGE

According to the working principles of BN/SDS-PAGE, proteins that are components of the same protein complex will appear in the same vertical line on the 2D BN-PAGE. Therefore, we analyzed the potential protein complexes based on the protein profile of the SDS-PAGE, and then the information from the corresponding BN-PAGE bands was taken into consideration. At least 34 potential heterooligomeric protein complexes were found in the SDS-PAGE (Table S3). Among them, thirty-three known multiprotein complexes were identified in our analysis. In addition, a new protein complex (clathrin and SK channel protein 2) was identified. All the protein complexes identified from Figure 2 were listed in Table S3.

### Validation of Novel Multi-protein Complexes by Antibody-based Gel Shift Assay, co-immunoprecipitation and Immunofluorescence

**Figure 4:**
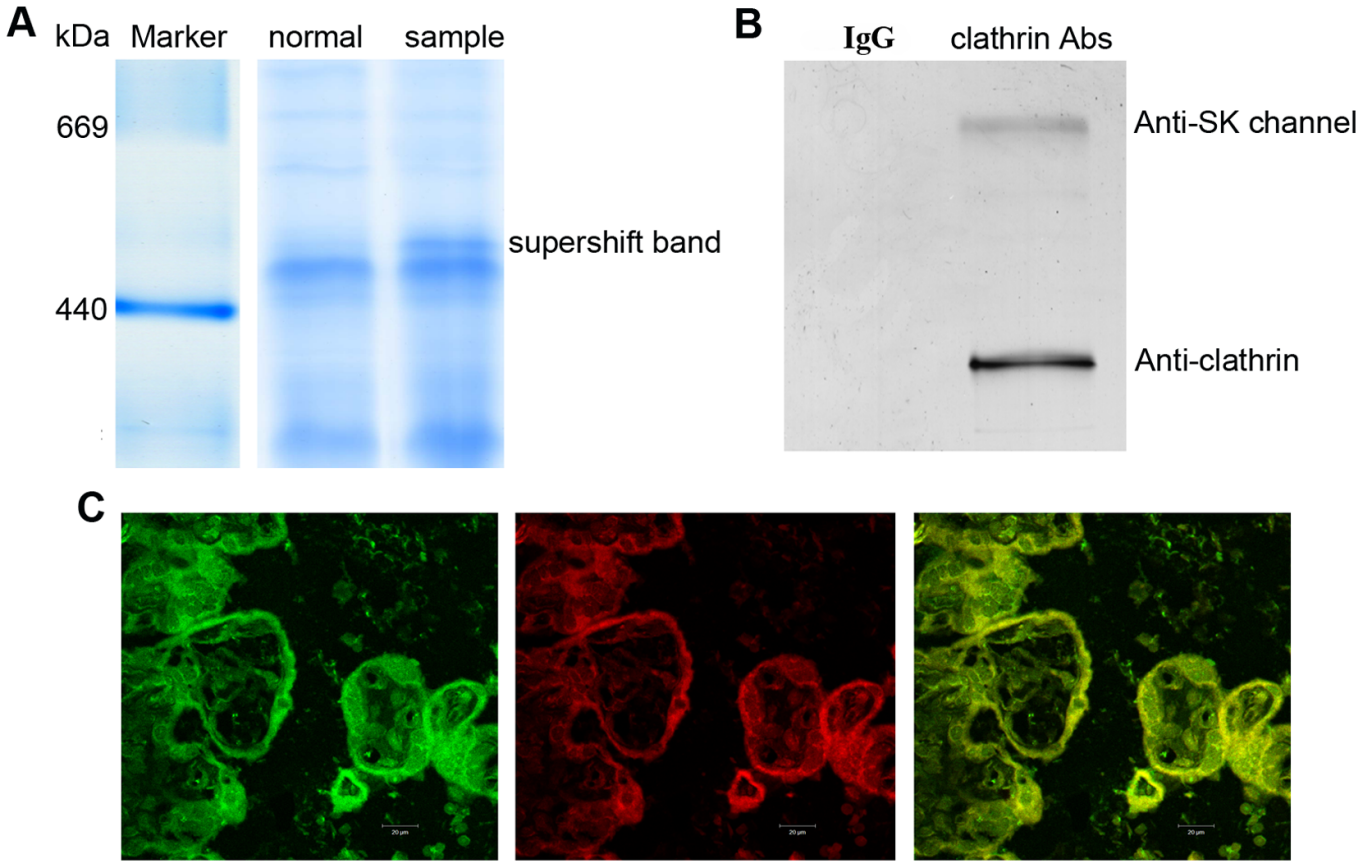
Verification of the validity of clathrin-SK channels protein complex. (A) Verification of the validity of the protein complex by BN-PAGE supershift assays. Placental lysates were incubated with SK channel protein 2 antibody (Ab) and resolved by BN-PAGE. A supershift band was indicated by arrow; no supershift was observed when lysates were incubated with normal IgG. (B) Copurification of clathrin and SK channel protein 2 protein complex from placental by co-immunoprecipitation. Placental protein lysates were either incubated with a clathrin-specific antibody (anti-clathrin) or a preimmunisation IgG pool (as negative control), then subject to SDS PAGE and western blot analyses. (C) Immunohistochemical analysis of the colocalization of clathrin and SK channel protein 2 in trophoblastic layer of placental. Immunohistochemical localization of SK channel protein 2 (left), clathrin (middle), and colocalization of clathrin and SK channel protein 2 (right).

As we discussed above, the positions of two protein spots in the same vertical line on a 2D BN/SDS gel indicate the possibility of their association in the same protein complex. However, due to the limited resolution of BN-PAGE, each protein could also be part of separate complexes that migrate to the same position on BN-PAGE. To prove the validity of the novel protein complex identified, we performed an antibody-based gel shift assay which was reported as a useful method to verify whether a cluster of proteins are in the same protein complex or not [21]. The protein lysates of placenta were incubated with anti-SK channel protein 2 antibody or normal IgG (negative control) prior to BN-PAGE. Figure 4A shows that pre-incubation of the protein lysates with an anti-SK channel protein 2 antibody resulted in the presence of a slower migrating complex compared with those incubated with normal IgG. To further characterize the components of supershift band, the band was cut and subjected for MS/MS analysis. The results indicate that the complex contains SK channel protein 2 and clathrin (Figure S1).

The association of these two proteins was confirmed by subsequent reverse copurification of SK channel protein 2 from placenta using a clathrin-specific antibody. As indicated by western blots, SK channel protein 2 was copurified by anti-clathrin but not by the pool of preimmunization IgGs (Figure 4B). Immunofluorescence staining of trophoblastic layer of placenta indicates that these two proteins co-localize in the trophoblast of placenta (Figure 4C). All of these results above suggest that SK channel protein 2 is associated with clathrin protein in placenta.

## Discussion

### Placenta Protein Complexes Identified by BN/SDS-PAGE

The placenta plays critical roles in many physiological functions including the exchange of key molecules necessary for fetal development and metabolism. Most of current techniques are used to study the protein profile of placenta on a proteome-wide scale. As we known, protein interactions are central to most biological processes. Only a few techniques such as BN-PAGE are available to visualize and identify the interaction of proteins *in vivo* on a proteome-wide scale. In this study, BN/SDS-PAGE was used to analyze the placenta proteins and protein complexes. In total, 733 unique proteins and 34 protein complexes were separated and identified.

The general interpretative rule for the analysis of the BN/SDS-PAGE is that all protein spots which are situated vertically are potential subunits of a same protein complex. However, due to the limitation of the resolution of electrophoresis system, more than one protein complex could be separated to the same molecular mass band during BN-PAGE and more than one protein subunit might be resolved to have same molecular mass. In other word, should not all protein subunits in the same vertical row do belong to same protein complex [22]. In our study, 34 unique protein complexes were identified, of which 33 were known protein complexes. One novel protein complex, clathrin and SK channel protein 2, was successfully identified.

To visualize protein-protein interactions, the protein complexes involved in energy generation were used as internal standards because these protein complexes have been identified and reported by other biochemical researches [17]. All of these previously characterized protein complexes involved in the mitochondrial respiratory chain were also identified in our research. Therefore, BN-PAGE is actually an effective tool for the isolation of protein complexes from placenta.

### Heteromeric Complexes Involved in Energy Generation

Mitochondria are key regulators of ATP production and their roles in energy production are fundamentally important during early oocyte and embryo development [23].The mitochondrial respiratory chain consists of multi-subunit protein complexes embedded in the inner membrane comprising complex I (NADH-ubiquinone oxidoreductase, EC 1.6.5.3), complex II (succinate-ubiquinone oxidoreductase, EC 1.3.5.1), complex III (ubiquinol-ferricytochrome c oxidoreductase, EC 1.10.2.2), complex IV (cytochrome c oxidoreductase, EC 1.9.3.1), and complex V (F1F0 ATPase) [24]. In this study, 19 subunits of complex I,3 subunits of succinate dehydrogenase, 13 subunits of complex IV,7 subunits of cytochrome b-c1 complex, and 11 subunits of complex V were identified by our method (Table S3). In addition, 4 isocitrate dehydrogenase [NAD] subunits and 2 subunits of electron transfer flavoprotein were also identified.

### Heteromeric Integrin Complexes

Integrins are heterodimeric transmembrane receptors composed of α and β subunits which were essential for adhesion of cells to the extracellular matrix. In addition, integrins also regulate the cellular phenotype in the developing and post-natal myocardium, modulate cell survival and migration, and remodel cellular cytoskeleton and focal adhesion [25]. During the differentiation of human trophoblast cells, a switch of integrin expression occurs which assists the trophoblast cells to acquire an invasive phenotype [26]. Research indicated that the α_1_β_1_ integrin complex plays a significant role in cellular interactions with interstitial collagen which are involved in matrix remodeling processes such as morphogenesis and wound healing [27]. In this study, 7 α-subunits and 4 β-subunits of intergrin in placenta were found and identified.

### Heteromeric Proteasome Complexes

In the eukaryotic cells, the ubiquitin-proteasome system (UPS) is of particular importance because it is involved in the selective degradation of the short-lived intracellular proteins [28]. During early pregnancy, the UPS participates in the degradation of the extracellular matrix (ECM) and trophoblastic invasion [29]. Proteasome complexes exhibit a high degree of heterogeneity in their overall subunit compositions and constitute dynamic structures in response to various cellular environments such as inflammation, the stage of development, or the pathophysiologic context [28], [30], [31]. Up to date, no precisely structural determination of the whole proteasome complex is available. In this study, 6 α-subunits and 9 β-subunits of proteasome in placenta were separated and identified.

### Heteromeric Histone Complex

Histones are highly positively charged proteins that wrap the genome. Whole histone extracted from chromatin by either acid or protamine displacement at pH 7 contains only two histone complexes, H2A-H2B and H3–H4. A systematic investigation of binary, ternary, and quaternary histone mixtures revealed that interactions also occur between histones H2B-H4 and H2A-H4 [32]. MAP3K4/CBP-regulated H2B acetylation controls epithelial-mesenchymal transition in trophoblast stem cells [33]. In this study, Histone H4, Histone H2A, and Histone H2B in placenta were identified as protein complexes.

### Heteromeric Heat Shock Protein Complexes

Oxidative stress has been increasingly postulated as a major contributor to endothelial dysfunction in preeclampsia (PE). The reactive oxygen species promote lipid oxidation and are known to induce stress proteins such as heat-shock protein 70 (HSP70) [34]. Heat shock proteins defend the cell against diverse external physiological stresses [35]. These proteins take an important part in protein complex formation, contribute to protein folding and extension, and function in protein transport between cell organelles, and etc [36]. Coimmunoprecipitation experiments have demonstrated that HSP60, HSP70, and HSP90 interact with each other in HepG2.2.15 cells, suggesting that these HSPs function together in complex pathway [37]. Our study also found and identified this important protein complex.

### Other Known Protein Complexes

The annexins are a family of calcium- and phospholipid-binding proteins that have been implicated in channel formation, membrane fusion, vesicle transport and regulation of phospholipase A2 activity [38]. It is reported that the presence of maternal annexin A5 minimizes the risk of thrombosis in the placental circulation and reduces the risk of foetal loss [39]. In this study, we found that annexin A2 interacts with caveolin 1. This complex (i.e., annexin A2 interacts and caveolin 1) had been found in the zebrafish and mouse intestine [40], but not in placenta. In addition, annexins have the requisite properties to integrate Ca^2+^-signaling with actin dynamics at membrane contact sites. Other researchers also found that annexins 1 and 2 harbour phosphorylation sites for different signal transducing kinases and binding sites for two EF hand Ca^2+^-binding proteins (S100A11 and S100A10) [41], [42]. In this study, annexin-actin and annexins 1- S100-A11 complexes were successfully found and identified.

During pregnancy, the fetus accumulate 25 to 30 g of calcium [43], which is transported across the STB cell layer from the mother to the child. Calcium transfer through the plasma membrane Ca^2+^-ATPase is the most important mechanism of Ca^2+^ homeostasis in the human placenta [44]. In this study, we identified 4 subunits of the Ca^2+^-ATPase (Ca^2+^-ATPase 1, 2, 3, and 4) from placenta.

### Clathrin and SK Channel Protein 2 Complex

Proteomic analysis of placenta by blue native PAGE shows that clathrin and SK channel protein 2 are assembled into one complex. Interaction between these two proteins was further validated by antibody-based gel shift assay, co immunoprecipitation, and immunofluorescence staining of placenta.

Clathrin-mediated endocytosis (CME) is a major mechanism the internalization of plasma-membrane receptors [45]. Clathrin coats vesicles to move molecules away from the plasma membrane [46]. SK channels are important voltage-independent ion channels which link intracellular calcium transients to membrane potential changes [47]. Our results indicate that clathrins might influence the number and subcellular localization of SK channels by promoting their traffickings to the plasma membrane. However, this conclusion needs more experimental evidence to support.

In conclusion, this study launched a comprehensive proteomics analysis of placenta protein complexes using 2D BN/SDS-PAGE and LC-MS/MS which resulted in the identification of 733 proteins and 34 protein complexes. For the first time, we found that clathrin associates with SK channel protein 2 to form a complex in placenta. These results suggest that BN/SDS-PAGE could be used to analyze functional protein complexes in placenta and applied to reveal the molecular mechanisms of pathological processes of the materno-fetal exchange.

## Supporting Information

Figure S1 MS/MS spectra of two peptides unique for SK channel protein 2 (A) and clathrin (B) obtained from the supershift bands in Figure 4A.
(TIF)

Table S1 Clinical characteristics of the patients included in this study.
(DOC)

Table S2 Placenta proteins identified by BN/SDS-PAGE.
(XLS)

Table S3 Placenta protein complexes identified by BN/SDS-PAGE.
(XLS)
