# Supplementary figures and images for: Identification and Analysis of Multi-Protein Complexes in Placenta
Source: PLoS One. 2013 Apr 29;8(4):e62988. doi: 10.1371/journal.pone.0062988 (PMC3639281; doi:10.1371/journal.pone.0062988)

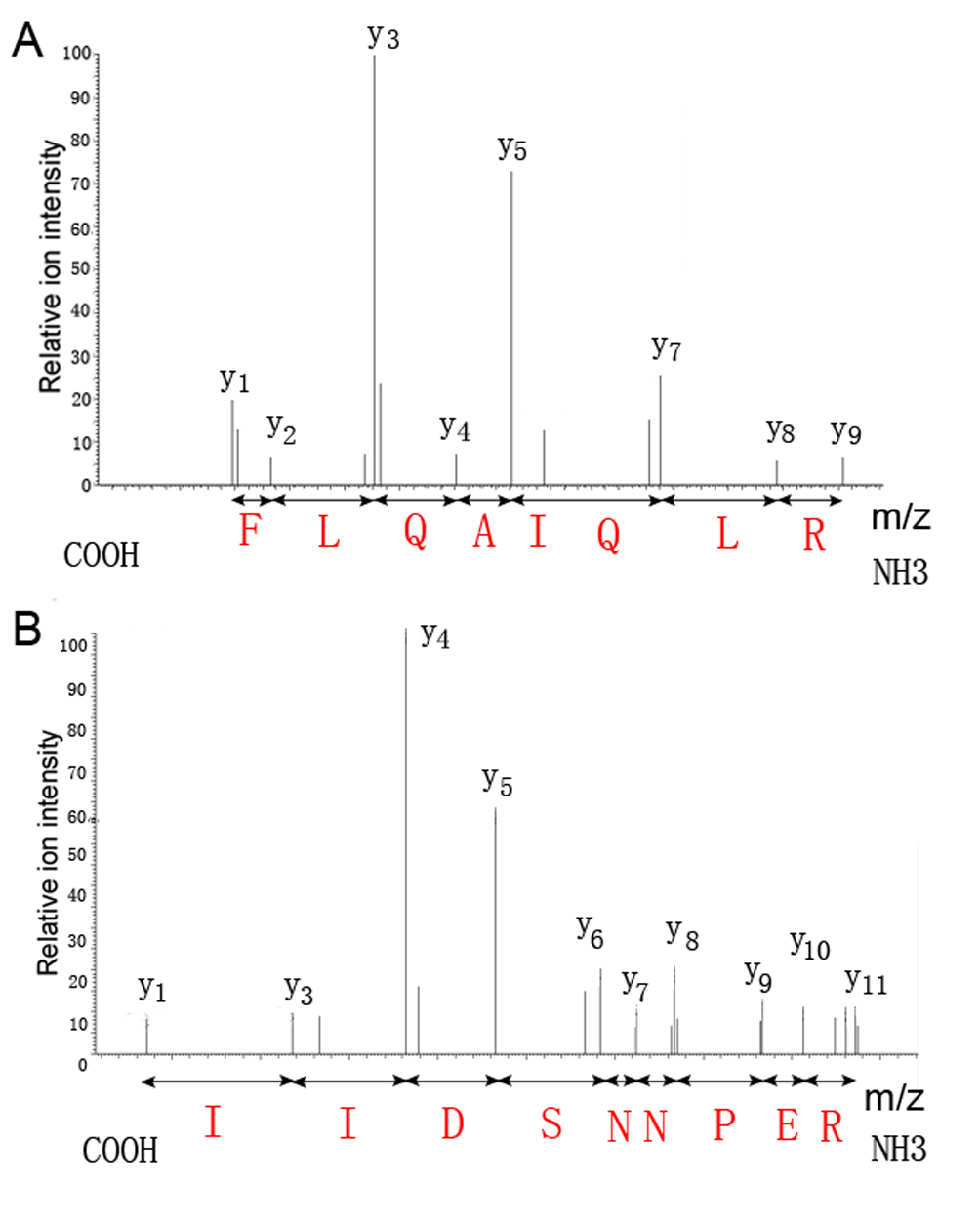

Supplement: Figure S1 — MS/MS spectra of two peptides unique for SK channel protein 2 (A) and clathrin (B) obtained from the supershift bands in Figure 4A. (TIF) [file pone.0062988.s001.tif]
