# Supplementary material for: Identification and Analysis of Multi-Protein Complexes in Placenta
Source: PLoS One. 2013 Apr 29;8(4):e62988. doi: 10.1371/journal.pone.0062988 (PMC3639281; doi:10.1371/journal.pone.0062988)
Supplement: Table S1 — Clinical characteristics of the patients included in this study. (DOC) [file pone.0062988.s002.doc]

**Table S1. Clinical characteristics of the patients included in this study**

|  | Controls (n = 20 ) |  |
| --- | --- | --- |
| Age (years) | 25.9 ± 2.7 |  |
| Gestational age (weeks) | 38.3 ± 2.8 |  |
| Manner of delivery | Caesarean section | |
| Systolic Blood pressure (mmHg) | 120.4 ± 6.3 |  |
| Diastolic Blood pressure (mmHg) | 72.1 ± 6.8 |  |
| Proteinuria (g/24h) | 0 |  |
| Platelets(×10^9^/L) | 239 ± 18 |  |
| ALT (U/l) | 24.1 ± 1.7 |  |
| AST (U/l) | 30.8 ± 3.5 |  |
| Birth Weight of infant (g) | 3524 ± 186 |  |
| Placental Weight (g) | 586 ± 193 |  |

Data are presented as mean ± SEM

^*^ *P* < 0.05 compared with control
